# Report of a Fatal Case of Hemophagocytic Lymphohistiocytosis Syndrome and a Review of the Literature

**DOI:** 10.7759/cureus.12049

**Published:** 2020-12-13

**Authors:** Hamza H Khan, Iqraa Ansar, Natalie Kontos, Sanjay Kumar, Hernando Lyons

**Affiliations:** 1 Pediatric Medicine, Ascension St. John Children’s Hospital, Detroit, USA; 2 Pediatric Medicine, Shifa International Hospital, Islamabad, PAK; 3 Pediatric Palliative Care, Ascension St. John Children's Hospital, Detroit, USA; 4 Pediatric Gastroenterology, Ascension St. John Children’s Hospital, Wayne State University School of Medicine, Detroit, USA

**Keywords:** epstein-barr virus, hemophagocytic lymphohistiocytosis (hlh), pancytopenia

## Abstract

Hemophagocytic lymphohistiocytosis (HLH) is a rare condition in children, with a high mortality rate of 41.99%. Often, due to the atypical presentation of HLH, the syndrome is difficult to diagnose. We report a case of an atypical presentation of HLH and the diagnostic dilemma that we faced. An 11-year-old boy was hospitalized with recurrent fever, hepatosplenomegaly, and worsening jaundice. Initial laboratory workup revealed an elevated prothrombin time (PT), high bilirubin, increased alanine transaminase (ALT), and positive Epstein Barr virus (EBV) deoxyribonucleic acid (DNA) polymerase chain reaction (PCR) and EBV immunoglobulin G (IgG). Based on our patient’s presentation and initial laboratory findings, further extensive workup was done, which revealed cytopenias, hypofibrinogenemia, hemophagocytosis on biopsy, absent natural killer (NK) cell activity, high serum ferritin level, and high soluble CD25 (sIL-2 receptor); a diagnosis of HLH was made. He was started on broad-spectrum antibiotics, antivirals, antifungals, and cyclosporine. He became hypoxic and hypotensive and hence was intubated and started on vasopressors. However, his coagulation profile continued to deteriorate. He started bleeding from multiple sites and became unresponsive to ventilatory support, eventually dying as a result of complications of HLH. The ambiguous clinical presentation makes the diagnosis of this syndrome difficult. However, due to the high fatality rate, early recognition and prompt treatment are of utmost importance.

## Introduction

Hemophagocytic lymphohistiocytosis (HLH) is a rare condition, with primary HLH having an incidence between 0.12 and 1 per 100,000 children [1-2]. The pattern of inheritance is autosomal-recessive, therefore, an increased incidence is seen in areas with high consanguinity [1-2]. Secondary HLH is more frequent than primary HLH but the data regarding incidence is sparse [3]. Machaczka et al. reported an incidence of 0.36 cases per 100,000 adults with malignancy-associated HLH [4]. Its incidence in North America is unknown [5].

HLH is characterized by fever, jaundice, hepatosplenomegaly, pancytopenia, and the characteristic histological features of hemophagocytosis in tissue and bone marrow biopsy [6]. HLH is clearly a rare entity with ambiguous clinical presentation and multiple differential diagnoses. Without treatment, the average survival of familial (or primary) HLH is approximately two months [1,7]. Since untreated HLH has a high mortality rate even with aggressive chemotherapy, a high index of suspicion is required for its early diagnosis and more effective treatment [8]. In this case, we discussed a rare presentation of HLH in order to refresh knowledge and improve awareness about clinical presentation.

## Case presentation

Our patient was an 11-year-old vaccinated boy from Senegal, Africa, who presented to the Ascension St. John Hospital's emergency department (ED) with complaints of fever, headache, and yellowish discoloration of the eyes for two weeks. He was born in the US but had been living in Africa for the past seven to eight years. The patient is a healthy child at baseline, with no significant past medical history, except for undocumented intermittent fever for the last six months, lasting two to three days each month. For the last two weeks, he had also developed scleral icterus, intermittent frontal headache, and decreased appetite without any associated visual changes, vomiting, or change in bowel movement. His viral hepatitis panel (including hepatitis A, B, and C) was negative, so he was discharged home on symptomatic treatment.

Eleven days later, the patient returned to the ED due to worsening jaundice and continued fever. At this visit, it was revealed that he was recently diagnosed with liver disease by a physician in Africa, but the exact details regarding the diagnosis were not available. There was no history of rash, fatigability, easy bruising, seizures, eating undercooked meat, water plants, or exposure to mosquitos. On examination, he was anxious but in no acute distress, alert and oriented, and vitally stable. His physical examination was significant for scleral icterus, hepatomegaly, and splenomegaly with a margin of 3 cm below the costal margins and firm in consistency without any nodularity. Table 1 summarizes his initial laboratory workup. He continued to spike fever to more than 39.0-degree centigrade every day.

**Table 1:** Laboratory workup

	Admission Day 1		Admission Day 2	Admission Day 7	
White Blood Cell (WBC)	5200/mm3	Neutrophils-43%		3200/mm3	absolute neutrophil count (ANC)- 1000/mm3
		Lymphocytes-37.6%			
		Eosinophil-9.1%			
		Monocytes-9.8%			
Hemoglobin (Hb)	10.4 gm/dl			8.7 gm/dL	
Hematocrit (Hct)	30.4%			25.2%	
Platelets (Plts)	219000/mm3			126000/mm3	
Complete Metabolic Panel					
Electrolytes	Normal				
Blood Urea Nitrogen (BUN)	Normal				
Creatinine (Cr)	Normal				
Aspartate aminotransferase (AST)	310 IU/L				
Alanine aminotransferase (ALT)	191 IU/L		1298 IU/L		
Alkaline phosphatase	198 IU/L				
Total bilirubin	2 mg/dl		3.9 mg/dl	9.1 mg/dl	
Direct bilirubin	1.1 mg/dl		2.2 mg/dl	7.1 mg/dl	
Miscellaneous					
Sickle cell screen	Positive				
Amylase			Normal		
Lipase			Normal		

Gastroenterology and infectious disease consultation were obtained. Given his origin from Africa, an extensive workup for infections was performed, including nasopharyngeal viral culture, blood culture, stool for ova and parasite, smear for the malarial parasite, antibodies for Epstein Barr virus (EBV), cytomegalovirus (CMV), measles, Varicella, human immunodeficiency virus (HIV), Lyme disease, Brucella, and Echinococcosis. Except for elevated CMV and EBV immunoglobulin G (IgG), all other tests were negative. Due to his history of recurrent fever every month, he was placed on penicillin for empirical treatment of relapsing fever. The gastroenterologist ordered a workup to evaluate the relevant causes. Table 2 summarizes the workup ordered by the gastroenterologist.

**Table 2:** The gastroenterologist workup ANA: antinuclear antibodies; PT: prothrombin time; PTT: partial thromboplastin time

Alpha 1 anti-trypsin	Normal
Ceruloplasmin	Normal
Sedimentation rate	64mm/hr
ANA	Negative
Liver microsomes antibodies	Negative
Smooth muscle antibodies	Positive (40)
PT	Prolong
PTT	Prolong

His prothrombin time (PT) and partial thromboplastin time (PTT) failed to correct with vitamin K (Vit K) injection. Subsequently, he was evaluated by hematology-oncology. A full coagulation workup was ordered that revealed that his fibrinogen levels were normal and D dimer was negative. PT was prolonged with mixing, whereas PTT got corrected after mixing. The factor X was low at 20%. With clearly worsening liver function, he was infused with fresh frozen plasma, and the decision to do liver and bone marrow biopsy was made.

The patient’s bone marrow exam revealed rare noncaseating granulomas and poorly formed lymphoid-histiocytic clusters accounting for < 1% of the volume. It showed 50%-60% cellularity with microcytic erythrocytes, but all other cell lines were normal. It did not demonstrate any infiltrative disorder. His peripheral smear demonstrated microcytic hypochromic red cells with mildly reduced leukocytes and platelets with normal morphology. Bone marrow did not have any acid-fast bacilli and was negative for any organisms. The fungal culture was negative too.

The liver biopsy was inconclusive, revealing prominent lobular inflammation with pseudo-rosette formation and plasma cell infiltration. A presumptive diagnosis of autoimmune hepatitis was made, and he was started on steroids. His fever subsided, and he was discharged home on steroids.

During his follow-up visit 1 week later, he showed significant improvement. His bilirubin had gone down from a pre-discharge value of 11.4 mg/dl to 3.7 mg/dl, and ALT had decreased to 296 units per liter. However, this improvement did not last long. At his next follow-up visit, his liver enzymes increased further. He was icteric again with a bilirubin total of 11.8 mg/dl and lethargic. He was transferred to the tertiary care center for further workup and management.

He continued to spike fevers intermittently at the other tertiary care center. His liver status continued to decline, and he developed coagulopathy. Table 3 summarizes the initial laboratory findings at the tertiary care center. 

**Table 3:** Initial laboratory findings at the tertiary care center

Absolute neutrophil count (ANC)	1.7 K/mm3		
Hemoglobin (Hb)	8.5 g/dL		MCV - 81.4 fl
Hematocrit (Hct)	24%		
Platelets (Plts)	60 K/mm3		
Liver Function and coagulation profile			
Fibrinogen		67 mg/dL	
Prothrombin time (PT)		35.2sec	
Partial thromboplastin time (PTT)		44.4sec	
International normalized ratio (INR)		3.6	
Alanine transaminase (ALT)		3763 units/l	
Miscellaneous			
Triglycerides		66 mg/dL	
Ferritin level		1445.6 ng/mL	
Leishmania antibodies		Negative	
Malaria parasite thick and thin smear		Negative	
Hepatitis (A, B, C)		Negative	

On the pediatric floor, the patient developed an acute cough episode associated with desaturation to 84%. He was tachycardiac and tachypneic, which did not respond to nasal oxygen, and, hence, he was transferred to the pediatric intensive care unit (PICU). His condition worsened, and he became hypoxic, agitated, disoriented, and hypotensive. Thus, he was intubated and placed on a conventional ventilator. He was started on epinephrine and milrinone to maintain his blood pressure, and acyclovir, cefepime, and doxycycline were added to his regimen for broad-spectrum coverage.

Table 4 summarizes the extensive workup for a possible infectious cause initiated at the PICU.

**Table 4:** PICU workup EBV: Epstein Barr virus; DNA: deoxyribonucleic acid; PCR: polymerase chain reaction; PICU: pediatric intensive care unit

Miscellaneous	
EBV DNA PCR blood	>30,000
EBV DNA PCR tracheal aspirate	<500
Soluble interleukin-2 receptor	7668 unit/ml
CD45+	<200
Natural killer cells	Absent
X-ray chest	Mild bronchial marking prominence
Ultrasound abdomen	Splenomegaly, non-specific thickening of gallbladder consistent with hepatitis
Bone marrow biopsy	Hemophagocytic histiocytes ingesting intact cells and cell debris
Syntaxin 11	Negative
MUNC 13-4	Negative
SH2D1A	Negative
Perforin 1	Negative

**Figure 1:**
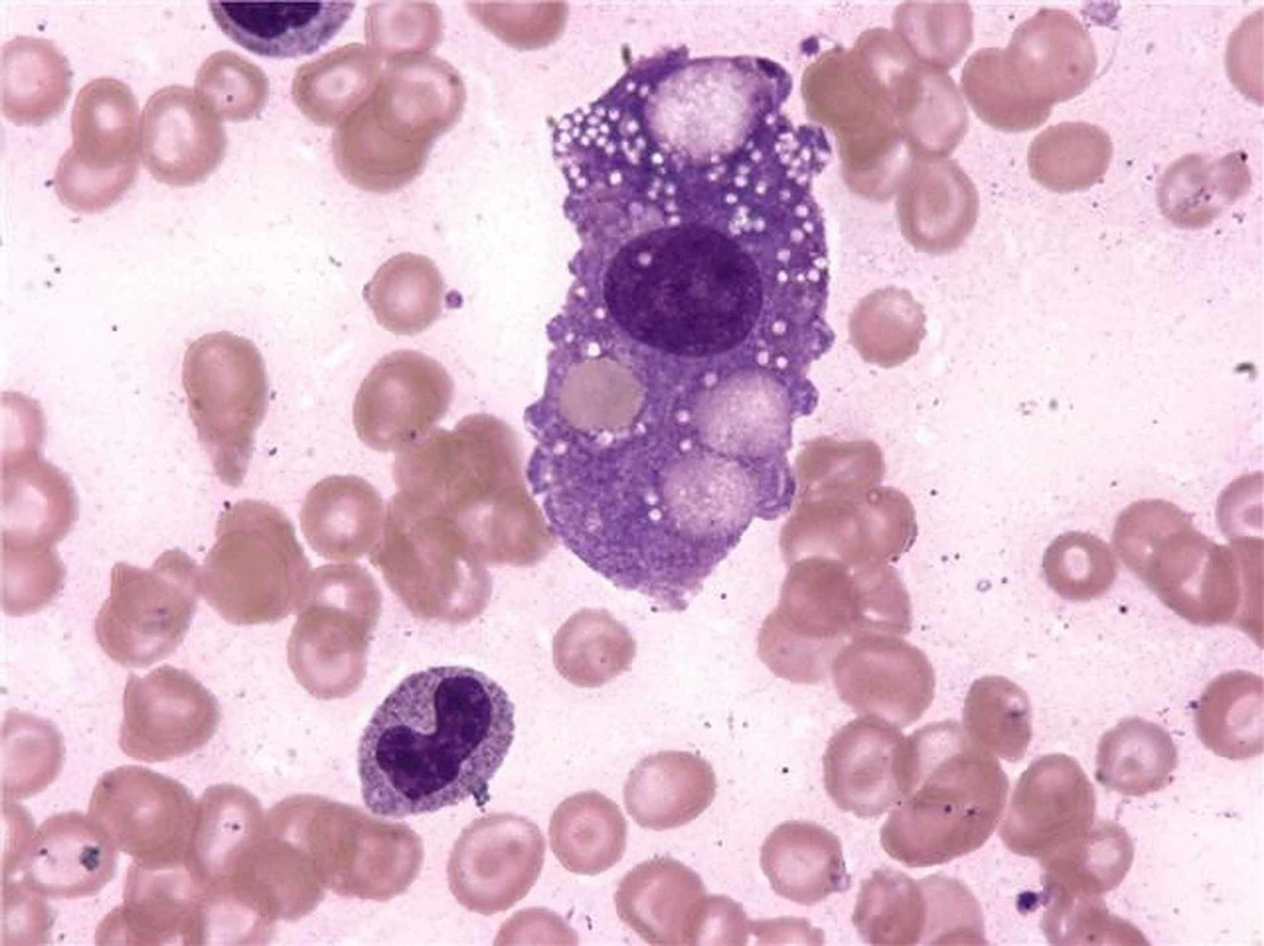
Hemophagocytosis-lymphohistiocytes showing the ingestion of intact cells and cell debris

Bone marrow biopsy findings (Figure 1), along with negative molecular genetic testing for familial hemophagocytic lymphohistiocytosis (FHL) confirmed the diagnosis of EBV-induced hemophagocytic syndrome with multiple organ failure.

He was continued on vasopressors to maintain his blood pressure, the rest of his regimen was revisited, and he was started on dexamethasone, cyclosporine, etoposide, etanercept, and ganciclovir. Fluconazole and cefepime were added for prophylaxis secondary to his cytopenias. Plasma exchange was done. His condition continued to deteriorate; his chest imagining studies were suggestive of acute respiratory distress syndrome (ARDS) with extensive pulmonary opacities. His white blood cells (WBCs), hemoglobin, and platelets dropped to 0.3/µL, 10.4 gm/dl, and 49K/µL, respectively. His bilirubin increased to 33.4 mg/dl and ammonia was 110 mcg/dL, whereas his alanine transaminase (ALT) and aspartate aminotransferase (AST) kept on declining.

He developed severe coagulopathy and was bleeding from the endotracheal (ET) tube, nasogastric (NG) tube, and suprapubic catheter that required multiple packed red blood cell (PRBC), platelet, and fresh frozen plasma (FFP) transfusions. In two days, he had to be placed on a high-frequency oscillator due to the inability to maintain appropriate saturations. Finally, he developed hypoxia unresponsive to ventilator support and succumbed to hemophagocytosis syndrome.

## Discussion

Hemophagocytic lymphohistiocytosis is a rare but potentially life-threatening disorder that goes by different names, including familial erythrophagocytic lymphohistiocytosis (FEL), viral-associated hemophagocytic syndrome (VAHS), and familial hemophagocytic lymphohistiocytosis (FHL). It is classified into “primary HLH” where there is an underlying genetic disorder and "secondary HLH," which represents the HLH phenomenon occurring secondary to an infection such as EBV, autoimmune disorders such as juvenile idiopathic arthritis, or malignancies including non-Hodgkin’s lymphomas (NHL) [2].

Viruses are the most common triggers for secondary HLH, and EBV (like in our patient) is the most common viral trigger [9]. Other viruses include CMV, HSV, HPV, HIV, parvovirus B19, and human herpesvirus (HHV) 8 [10-11]. Similar to our case, Gowarty et al. reported a case of hemophagocytic lymphohistiocytosis, where the patient was found to be positive for EBV. In addition, the patient was positive for parvovirus B19. They treated him with steroids, which resulted in remarkable improvement [12]. Among the bacterial causes, tuberculosis and gram-negative organisms have been implicated [10-11]. Parasites, including Plasmodium and Leishmania, and fungal infections, such as Candida and Cryptococcus, have been known to play a role too [10-11].

Cytokine dysfunction has been described as the main pathophysiological abnormality leading to excessive buildup of activated histiocytes (macrophages) and T-lymphocytes in many organs [8,13-14]. Interleukin-2 (IL-2) and IL-18 have been the main ones implicated [6]. Levels of soluble IL-2 receptor antibodies were elevated in our patient to 7668 (normal levels - 0-5 pg/ml). Our patient had high levels of immunoglobulin levels (3,590 mg/dl), but immunoglobulin levels are usually normal in these patients. The cytotoxic T cell and natural killer (NK) cell activity is decreased in patients with HLH [6]; NK cells were absent in our patient.

Gene defects in FHL2-Perforin (PRF1), FHL3-Munc13-4 (UNC13D), and FHL4-Syntaxin11 (STX11) have been linked to familial HLH [15-17]. Protein defects involving the MUNC13-4 protein and RAB27A protein have been detected [15-17]. The MUNC13-4 protein plays a vital role in the packaging of the cytolytic granzymes and the RAB27A protein is important for the release of these lytic granules [15-17]. Defective apoptosis is another cellular defect in HLH, as activated lymphocytes in some patients with HLH are found to have decreased spontaneous activation of caspase-3-like enzyme activity [6]. Our patient was evaluated for the Syntaxin 11 (STX-11), MUNC13-4, SH2D1A, and Perforin 1 (PRF1) genes, which did not reveal any demonstrable evidence of FHL.

X-linked lymphoproliferative (XLP) syndrome, a disorder seen almost exclusively in males, is also associated with HLH. XLP is due to a mutation at Xq25 that results in an abnormal immune response to EBV. This results in an unregulated overwhelming increase in macrophages, cytotoxic killer cells, and EBV-infected B cells [18]. It can be of two types, XLP1(SAP deficiency) and XLP2(XIAP deficiency). XLP1 has variable clinical presentations, including fatal HLH secondary to EBV [19] as seen in our patient; however, our patient tested negative for SAP mutation, and hence XLP1 was excluded. XLP2 causes recurrent HLH with or without prior EBV infection [19]. Our patient did not haVe a history of recurrent HLH.

The clinical features of HLH mimic other common clinical conditions like viral hepatitis, other viral illnesses, and fever of unknown origin (FUO) [6]. Fever and hepatosplenomegaly are found in a large number of patients with HLH [6]. Our patient’s six-month history of recurrent fever was confusing and made the diagnosis difficult, and his origin from Africa led us towards considering an infectious cause. The radiologic and clinical findings of many of these patients resemble acute respiratory distress syndrome, with pleural effusions and alveolar-interstitial opacities, as was seen in our patient’s CT scan. Ultrasonography may show gall bladder wall thickening (which was seen in our patient), hyperechoic kidneys, ascites, and hepatosplenomegaly.

Current diagnostic criteria for HLH are based upon the criteria used in the HLH-2004 trial [20]. The diagnostic criteria include either:

1. Molecular identification of gene mutation associated with HLH.

OR

2. The presence of five of the following eight findings.

(a) Seven or more days of peak temperature > 38.5 degrees C.

(b) A palpable splenomegaly of >3 cm below the left costal margin.

(c) More than one cell lines with cytopenias: Hemoglobin <9.0 g/dL, or Platelets <100,000/µL, or Absolute neutrophil count <1,000/µL.

(d) Either hypofibrinogenemia or hypertriglyceridemia: Hypertriglyceridemia with a value >3 standard deviation (SD) above age-appropriate normal value or > 2.0 mmol/L for fasting triglycerides. Hypofibrinogenemia with a value > 3 SD below age-appropriate normal value or fibrinogen level of <1.5 g/L.

(e) Hemophagocytosis, seen in the lymph node, bone marrow, or spleen, without any evidence of malignancy. Repeated attempts to identify the characteristic histology in lymph node biopsy or bone marrow aspirates may be necessary.

(f) Absent or low NK cell activity.

(g) >500 µg/L serum ferritin level.

(h) >2400 U/mL soluble CD25 (sIL-2 receptor).

During the initial admission of our patient, PT was prolonged with mixing, whereas PTT got corrected after mixing. This and the fact that they did not improve after vitamin K and fresh frozen plasma (FFP) suggested an inhibitor rather than coagulation factor deficiency. The low factor X (20%) suggested an anti-factor X inhibitor specifically. The microcytosis was consistent with iron deficiency anemia, as shown by the depleted iron stores on the Prussian blue stain.

During this same admission, given his elevated immunoglobulin, positive smooth muscle antibody, and liver biopsy findings, a presumptive diagnosis of autoimmune hepatitis was made though an acute viral or toxic hepatitis could not be excluded. He was started on steroids, to which the fever responded, and he was discharged home. Azathioprine (Imuran) was not started in addition to steroids since there was not a clear-cut diagnosis of autoimmune hepatitis, and we decided not to add more immunosuppressants.

Of note, over the course, our patient had fevers, splenomegaly, hemoglobin (Hb) <9 g/dL, platelets (Plts) < 100,000/µL, hypofibrinogenemia, hemophagocytosis on biopsy, absent NK cell activity, >500 µg/L serum ferritin level, and >2400 U/mL soluble CD25 (sIL-2 receptor) suggestive of primary HLH. However, there is a possibility that our patient developed secondary HLH i.e. VAHS (in this case), as the initial workup was positive for CMV and EBV IgG; the viral infection could have been reactivated with the steroids treatment that the patient received for suspected autoimmune hepatitis.

Treatment should be initiated promptly after the diagnosis. Since delays in appropriate treatment initiation can be fatal, treatment should be started if there is a high clinical suspicion, even if the patient meets only four of the diagnostic criteria. In the appropriate clinical setting, the diagnosis is justified by a positive family history of HLH; parental consanguinity is only suggestive of HLH.

Treatment, according to the HLH 2004 protocol, includes induction therapy with dexamethasone, cyclosporine, etoposide (VP-16), and intrathecal methotrexate, followed by pulses of dexamethasone and VP-16 for up to one year [20]. Heterologous bone marrow transplant is an option for patients with central nervous system (CNS) disease, for those with homozygous mutations in HLH-associated genes, and for patients who responded poorly to the initial eight weeks of therapy [20].

## Conclusions

We conclude that HLH has a variable presentation and is difficult to diagnose. The importance of early identification and prompt treatment can be inferred from our case. Although our patient was treated with dexamethasone, cyclosporine, etoposide, etanercept, and plasma exchange in addition to gancyclovir for EBV infection, the treatment had little impact since the early atypical presentation made the diagnosis difficult and the initiation of appropriate treatment was delayed.
